# 

**Published:** 1899-08-12

**Authors:** 


### NOTICE TO CORRESPONDENTS.

All MS., Letters, Books for Review, and other matters intended for the Editor should be addressed The Editor, "The Hospital," The Hospital Building, 28 & 29, Southampton Street, Strand, W.C.
The Editor cannot undertake to return rejected MS., even when accompanied by stamped directed envelope.

**Notice to Advertisers and Subscribers.**

All Advertisements, Orders for Copies of The Hospital, and Business Communications should be addressed to **THE MANAGER (Not to the Editor)**, The Hospital, The Hospital Building, 28 & 29, Southampton Street, Strand, London, W.C.
The Cost of Subscription, post free, is as follows:—Per week, 2½d.; per quarter, 2s. 8d.; per half-year, 5s. 3d.; per annum, 10s. 6d. Foreign:—Per annum, 15s. 2d., payable, in all cases, in advance.

**NOTICE.—Vol. XXV. of "The Hospital" (October, 1898, to March, 1899), in handsome cloth binding, is now on sale at the Office of this Journal, price 7s. 6d. Cases for binding the Numbers contained in this Volume 1s. 6d. Index to Vol. XXV., 2½d., post free.**

**The Monthly Part for August is now ready. Price 6d., by post 8d.**

### The Student of Medicine.

### APPOINTMENTS.

T. B. Broadway, M.B., Ch.B.Glasg., appointed Medical Officer for the Second District of the St. Austell Union. J. Butler, M.B., C.M.Glasg , appointed Senior Assistant Medical Officer to Govan District Asylum, Hawkhead. W. F. Byford, M.R.C.S.. L.R.C.P.Lond., appointed Medical Officer for the No. 1 Llanfair District of the Ruthin Union. C. A. Corke, L.F.P.S.Glasg., L.R.C.P.Edin., appointed Medical Officer of Health to the Pebworth Rural District. E. F. N. Currey, M.R.C.S.Eng., L.R.C.P.Lond., appointed Medical Officer and Public Vaccinator to the Sheldon District, Honiton Union. J. G. Forbes, M.B., B.C.Camb., appointed Second Assistant Medical Officer of the Shoreditch Infirmary. R. Gooding, A.B.Lond., M.D., M.B., M.R.C.S., appointed Medical Officer of Health of the Barnstaple Port Sanitary District. C. Hicks, L.R.C.P.Edin., L.R.C.S Edin., L.F.P.S.Glasg., L.M., appointed Medical Officer of the Seventh District of the Tonbridge Union. J. B. Hughes, M.R.C.S.Eng., L.S.A., appointed Senior Assistant Medical Officer to the Toxteth Park Workhouse. P. Kinmont, M.B., Ch.B.Edin., appointed a Resident Medical Assistant, Dundee Royal Infirmary. E. Pitway, L.S.A., appointed Medical Officer of the First District of the Saffron Walden Union. H. H. Roberts, M.B., Ch.B., appointed Medical Officer to Bury Tinplate Works, Llanelly. H. R. Spackman, F.R.S.P.H., appointed Medical Officer of Health to the Seisden Rural District. W. C. Sprague, M.D.Edin., L.R.C.P. Lond., M.R.C.S.Eng., appointed Medical Officer of the Seventh District of the Saffron Walden Union. J. E. Webb, M.B., C.M.Aberd., appointed Medical Officer of Health of the Love Urban District.

### ROYAL ARMY MEDICAL CORPS.

List of successful candidates for commissions in the Royal Army Medical Corps at the recent examination in London, arranged in order of merit: L. W. Harrison, 2,875 marks; F. S. Irvine, 2,284; H. M. Morton, 2,260; M. H. Babington, 2,231; F. G. Richards, 2,150; E. B. Knox, 2,121; H. T. Roch, 2,115; F. Harvey, 2,102; C. E. Trimble, 2,086; J. Matthews, 2,084; W. A. McLoughlin, 1,940; E. W. Siberry, 1,816; B. F. Wingate, 1,805; and P. S. O'Reilly, 1,800.

---

Paper set in Medicine and Pathology at the examination of candidates for the Royal Army Medical Corps and Her Majesty's Indian Medical Service on Friday, July 28th, 1899.

1. A man, æt. 36, had for some months been below par, and was losing flesh and colour, but continued at work, until one day, when he rapidly became comatose. On recovering consciousness it was found that his right arm and leg were completely, while the lower segment of the face on the same side was partially, paralysed, and to every question he returned for answer either "Yes" or "No." His heart was not sound, but there were no murmurs, and dropsy was absent. He had never had syphilis.

Fill in the picture of all the additional symptoms which might be present. Give the diagnosis in full, and what would you find *post mortem* in the event of a fatal issue?

2. Give an account of the indirect (pressure) symptoms which may be encountered in cases of aneurism of the arch of the aorta.

3. Give a short sketch of the complications of diabetes mellitus.

4. How can you satisfy yourself (*a*) that pus is present in the urine, (*b*) that it comes from the pelvis of the kidney, and (*c*) how would you treat the condition?

### Scraps and Gleanings.

After some discussion, the Liverpool Hospital Saturday and Sunday Fund have decided to make no grant this year to the Cancer Hospital.

The Scandinavian community at Cardiff have subscribed £111 towards the Seamen's New Hospital at Cardiff, making £158 received from this committee.

During last year the number of in-patients treated at the Children's Hospital, Great Ormond Street, was 2,007, and about 1,700 out-patients are treated weekly.

The amount of workpeople's contributions to the Swansea Hospital during 1898 was £1,320, being the highest sum yet reached, and an advancein seven years of nearly £1,000.

During the 34 years that the Belfast Hospital for Diseases of the Skin has been established 32,994 patients have been treated, the total number treated during the last year being 865.

The east wing of the Essex and Colchester Hospital, which has been thoroughly renovated, is now in working order. About half of the building is now nearing completion, and the cost of the whole work will be not much under £2,000.

At the annual meeting of the Guildford Cottage Hospital, held on August 3rd, the resignations of four of the honorary medical staff will be announced. These four resignations have been rendered necessary on account of a difference with the committee.

Owing to the growth of Coventry, the hospital accommodation there has become far too scant for the requirements of the inhabitants. The average number of inmates now is 70, while a few years back it was only 50, and accommodation for at least 20 more patients is urgently needed.

Of the £11,000 still required to complete the Preston Jubilee Infirmary Scheme, £6,000 is needed immediately to enable the improvements recommended by the Board of Management to be put in hand at once. These immediate improvements are to cost £14,000, towards which £8,000 has been collected.

At the twenty-fifth Annual Meeting of the Woolton Convalescent Institution a comparison was made of the work done during the past quarter of a century. The statistics show that whereas in 1878 there were 688 patients, with annual subscriptions amounting to £110, in 1898 there were 2,667 patients, with £898 in annual subscriptions. During 1898, £1,734 odd was paid by or on behalf of patients.

### Official Announcement.

HOSPITAL WARDER REQUIRED for the VICTORIA GAOL, HONG KONG.

Appointment for the first three years on probation.

Salary 720 dollars per annum, rising, by annual increments of 48 dollars, to 960 dollars. The salary is subject to a deduction of 4 per cent. to the Widows and Orphans' Pension Fund. Compensation allowance calculated to make up the difference between the current rate of exchange and 3s. to the dollar on half the salary will be granted. The present rate of exchange of the dollar is 1s. 11½d. Quarters, Fuel, Light, Servants, Uniform, and Medical Attendance.

Pension obtainable at the rate of 15/60 for the first ten years, with an addition of 1/60 for each succeeding year's service, on attaining the age of 55 years, or, in the event of retirement on the ground of ill-health, provided that at least ten years' service have been completed.

A free passage out provided.

Half salary from date of leaving England; full salary from date of arrival in the Colony.

A bounty of £5 will be paid on engagement.

Candidates must have had previous training in hospitals, preferably in the Army Medical Service Corps, and must be under 35 years of age and unmarried, and must furnish good references as to character and capability.

A strict medical examination will be required.

Applications, marked "Application for Hospital Warder, Hong Kong," addressed to the Crown Agents for the Colonies, Downing Street, London, S.W., will be received up to and inclusive of 19th August, 1899. (1,236)

